# Diagnostic accuracy and validation of ^18^F-fluorodeoxyglucose positron emission tomography scores in a large cohort of patients with polymyalgia rheumatica

**DOI:** 10.3389/fmed.2022.1026944

**Published:** 2022-09-21

**Authors:** Lien Moreel, Lennert Boeckxstaens, Albrecht Betrains, Maarten Van Hemelen, Steven Vanderschueren, Koen Van Laere, Daniel Blockmans

**Affiliations:** ^1^Department of General Internal Medicine, University Hospitals Leuven, Leuven, Belgium; ^2^Department of Microbiology, Immunology, and Transplantation, KU Leuven, Leuven, Belgium; ^3^Department of Nuclear Medicine, University Hospitals Leuven, Leuven, Belgium; ^4^European Reference Network for Immunodeficiency, Autoinflammatory, Autoimmune and Pediatric Rheumatic Disease (ERN-RITA), Utrecht, Netherlands; ^5^Department of Imaging and Pathology, Nuclear Medicine and Molecular Imaging, KU Leuven, Leuven, Belgium

**Keywords:** polymyalgia rheumatica (PMR), PET, diagnostic accuracy, validation, leuven score, Leuven/Groningen score

## Abstract

**Background:**

Several studies have shown that ^18^F-FDG PET may contribute to the diagnosis of polymyalgia rheumatica (PMR). Previously, we developed a composite PET score called the Leuven score, which was recently adapted to the more concise Leuven/Groningen score by van der Geest et al. The aim of this study is to validate and compare the diagnostic accuracy and cut-off points of both scores in a large cohort of PMR patients.

**Methods:**

Patients with a possible clinical diagnosis of PMR and a PET scan prior to the initiation of glucocorticoids between 2003 and 2020 were included retrospectively. The gold standard for the diagnosis of PMR was the judgment of two experienced clinicians after a follow-up of at least 6 months. FDG uptake was scored visually in 12 articular regions (scores 0–2) and a total skeletal score was calculated by summing the individual scores (maximum of 24 for the Leuven score and 14 for the Leuven/Groningen score). Receiver operating characteristic (ROC) analysis and the Youden index were used to determine the diagnostic accuracy and optimal cut-off points.

**Results:**

A total of 162 patients with PMR and 83 control patients were included. Both PET scores showed high diagnostic accuracy in the ROC analysis (area under the curve 0.986 and 0.980, respectively). The Leuven Score provided a sensitivity of 91.4%, specificity of 97.6% and accuracy of 93.5% at its predefined cut-off point of 16. With the newly determined cut-off point of 12 the sensitivity was 98.8%, the specificity 95.2% and the accuracy 97.6%. The Leuven/Groningen score had a sensitivity, specificity and accuracy of 93.2%, 95.2%, and 93.9%, respectively, with the pre-specified cut-off point of 8, and 96.9%, 92.8%, and 95.5% with the optimal cut-off point of 7.

**Conclusion:**

The original Leuven score and the simplified Leuven/Groningen score both had excellent diagnostic accuracy. The latter may be easier to apply in clinical practice.

## Introduction

Polymyalgia rheumatica (PMR) is a systemic inflammatory disease that affects elderly people. It is characterized by pain and morning stiffness in the neck, shoulders and pelvic girdle. It is commonly associated with constitutional symptoms and elevated inflammatory markers (1).

The diagnosis of PMR may be challenging, since there are no symptoms, laboratory abnormalities, or imaging findings specific for PMR. Other conditions, such as other musculoskeletal disorders, infectious disease and malignancy, should be ruled out. The 2012 EULAR/ACR criteria were developed for research and not for diagnostic purposes (2). In addition, the sensitivity and specificity of these criteria are quite low (68 and 78%, respectively).

**FIGURE 1:**
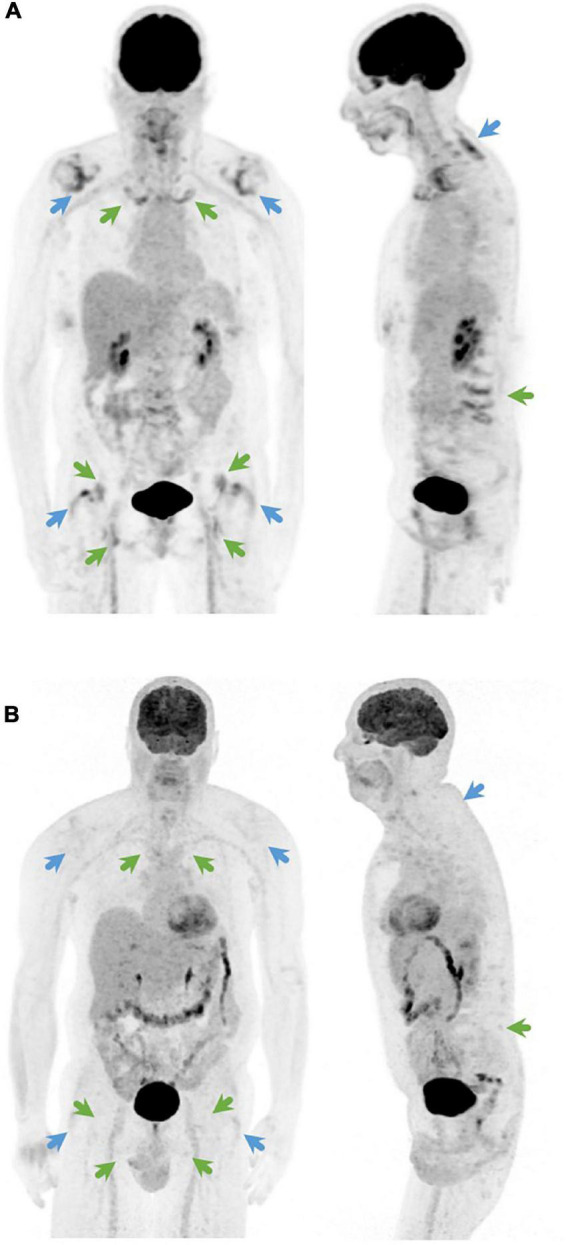
Coronal (left) and sagittal (right) ^18^F-FDG-PET image of **(A)** a PMR patient with a Leuven score of 24 and a Leuven/Groningen score of 14 and **(B)** a patient with fibromyalgia as final diagnosis with a Leuven score of 3 and a Leuven/Groningen score of 1. The Leuven score is the sum of joints indicated by green and blue arrows. The Leuven/Groningen score is the sum of joints indicated only by green arrows.

Several studies have shown that ^18^F-fluorodeoxyglucose (FDG) positron emission tomography (PET) may contribute to the diagnosis of PMR (3–6). FDG uptake is typically located at the shoulder and hip girdle, sternoclavicular joints, cervical and lumbar interspinous bursa, trochanteric bursa, and ischial tuberosities (4). Since individual sites do not provide sufficient diagnostic accuracy (7), several PET/CT algorithms (5, 8) and composite scores (4, 9–13) have been developed to determine when a PET/CT should be considered compatible with PMR. Direct comparison of 5 of these algorithms and composite scores (4, 5, 8, 11) showed that the Leuven Score, as reported by our group in 2018, had the best diagnostic accuracy with a sensitivity of 89.7% and specificity of 84.2% at the optimal cut-off point of 16 (14). Van der Geest et al. developed a more concise score, which is called the Leuven/Groningen score, based on the anatomic sites with an AUC ≥ 0.8 in ROC analysis (Figure 1) (14). This score provided a similar diagnostic accuracy with a sensitivity of 89.7% and specificity of 84.2% at the optimal cut-off point of 8.

In this study, we aim to validate and compare the diagnostic accuracy and cut-off points of the Leuven score and the Leuven/Groningen score in a large cohort of PMR patients.

## Materials and methods

### Patient population

We retrospectively included patients with a possible clinical diagnosis of PMR, who were evaluated by the Department of General Internal Medicine of the Leuven University Hospitals between 2003 and 2020 and had undergone PET imaging prior to the initiation of glucocorticoids. Patients with clinical symptoms of giant cell arteritis (GCA) or a positive temporal artery biopsy were excluded. Patients included in the study of Henckaerts et al., which were diagnosed between August 2012 and November 2015, were also excluded (4). The gold standard for the diagnosis of PMR was based on the judgment of two experienced clinicians (D.B. and S.V.) after at least 6 months of follow-up, considering all available information (clinical data, biochemical and radiological results, PET images, and evolution during follow-up).

The study was conducted in accordance with the Declaration of Helsinki and approved by the ethical committee of the University Hospitals Leuven. Informed consent was waived because of the retrospective nature of the study and the analysis used pseudonymized clinical data.

### Data collection

We collected the following patient data from the electronic health record: age, sex, date of diagnosis, duration of symptoms until PET, clinical symptoms at diagnosis (fever [defined as temperature ≥38.3°C], anorexia, weight loss, morning stiffness, shoulder and pelvic girdle pain, neck and lower back pain, pain and swelling of peripheral joints), laboratory values (erythrocyte sedimentation rate [ESR; mm/h], C-reactive protein [CRP; mg/L], hemoglobin [g/dL] and alkaline phosphatase [U/L]), and final diagnosis.

### Positron emission tomography imaging and analysis

Patients were required to fast for at least 6 h before intravenous injection of 4.5 MBq/kg of ^18^F-FDG, and glycemia levels were determined in all patients (<140 mg/dl). A whole-body PET scan was performed 45–60 min after tracer administration. PET scans were performed between 2003 and 2020, consecutively acquired on a ECAT HR + PET camera, Hirez Biograph 16 PET/CT or Truepoint Biograph 40 PET/CT (Siemens, Knoxville, TN, USA). Because of scan duration, HR + data were not corrected for attenuation using transmission scanning. On the PET/CT systems, either a low-dose non-diagnostic CT scan or a diagnostic CT scan with oral and intravenous contrast was performed immediately before PET acquisition.

Non-attenuation corrected (non-AC) PET images were available for all included patients and attenuation-corrected (AC) PET images were only available for the patients who were scanned with a PET/CT system (*n* = 119/245). PET data were corrected for scatter and randoms. Data were reconstructed using iterative OSEM reconstruction, with parameters optimized over the years (FWHM HR + 6–7 mm, for the Truepoint 5 mm).

Reconstructed PET images were re-evaluated visually by two independent specialists in nuclear medicine (K.V.L., L.B.), who were blinded for all other patient information. Both the non-AC PET images and the AC PET images were evaluated independently when available. To assess inter-reader reliability, PET images for the first 20 patients were evaluated by both specialists. The interrater agreement was assessed *via* the intra-class correlation coefficient (ICC) with a two-way mixed effects model. All other PET images were randomized and scored by one of both specialists only as interrater agreement was high in the sample set.

FDG PET uptake was visually assessed for 12 predefined skeletal regions (cervical spinous processes, lumbar spinous processes, left and right sternoclavicular joint, left and right ischial tuberosity, left and right greater trochanter, left and right hip, and left and right shoulder), and scored using a three-point scoring system: 0 (no elevated FDG uptake), 1 (moderately elevated FDG uptake, but less than mean liver uptake) or 2 (intense FDG uptake, equal or more than average liver uptake). The mean liver uptake is harder to assess in non-AC images as a radial non-linear gradient is present in the liver due to attenuation. The skeletal scores of the non-AC PET images and the AC PET images were compared if they were both available. The Leuven score was calculated for every patient, by summing the individual scores at the 12 different skeletal sites (total score of 0–24) (Figure 1). For the Leuven/Groningen score the scores for the lumbar spinous processes, sternoclavicular joints, ischial tuberosities and hips were summed with a total score ranging from 0 to 14.

### Statistical analysis

Categorical and continuous variables were expressed as count (percentage) and median ± interquartile range (IQR), respectively. Chi square tests or Mann-Whitney *U*-tests were used to compare characteristics between PMR patients and patients in whom PMR was initially suspected, but who received an alternative diagnosis after further tests and follow-up (further called “non-PMR patients”). The Leuven and Leuven/Groningen score were compared via the Mann-Whitney *U*-test. The sensitivity, specificity, accuracy, positive and negative likelihood ratio (LR) of the Leuven score and the Leuven/Groningen score were calculated for different cut-off values and plotted in a receiver operating characteristic (ROC) curve. The optimal cut-off value was determined by the Youden index (formula sensitivity + specificity–1). We did a sensitivity analysis excluding patients with only non-AC PET images. All statistical tests were performed using 2-tailed tests with significance set at the *p* < 0.05 level. Statistical analysis was performed in R Studio (version 2022.03.10, The R Foundation for Statistical Computing) with inclusion of the *psych, epiR, ggplot*, and p*ROC* packages.

## Results

A total of 245 patients were included in this study, of which 162 had a diagnosis of PMR and 83 received an alternative diagnosis (Supplementary Table 1). PET contributed to the alternative diagnosis in 8 non-PMR patients (9.6%): a paraneoplastic syndrome in 4 patients and dermatomyositis, polyarteritis nodosa, cholangitis and a liver abscess in one patient each.

The baseline characteristics are presented in Table 1. PMR patients were older compared to non-PMR patients (71 vs. 63 years, *p* = 0.001), but had a similar female to male ratio (55 vs. 51% female patients, *p* = 0.52). The symptom duration was significantly shorter in PMR patients (10 vs. 15 weeks, *p* = 0.02). PMR patients more frequently reported morning stiffness (89 vs. 69%, *p* = 0.003) and pelvic (82 vs. 66%, *p* = 0.006) and shoulder girdle pain (93 vs. 75%, *p* = 0.0001). There were no significant differences in neck and lower back pain, in pain and swelling of peripheral joints and in constitutional symptoms. ESR (47 vs. 31 mm/h, *p* = 0.007), CRP (36.0 vs. 15.8 mg/L, *p* = 0.0003) and alkaline phosphatase (134 vs. 86 U/L, *p* < 0.0001) were significantly higher in PMR patients compared to non-PMR patients. Hemoglobin was significantly lower (12.4 vs. 13.0 g/dL, *p* = 0.03).

**TABLE 1:** Baseline characteristics for the entire population.

Characteristics	Total (*n* = 245)	PMR (*n* = 162)	Non-PMR (*n* = 83)	*P*-value
Age at inclusion, years, median (IQR)	70 (60–76)	71 (63–77)	63 (56–75)	**0.001**
Sex, no. of females, *n* (%)	131 (53)	89 (55)	42 (51)	0.52
Symptom duration until PET/CT, weeks, median (IQR)	11.5 (5–25.75)^28^	10 (4.5–19)^16^	15 (6–41.5)^12^	**0.02**
**Symptoms, *n* (%)**				
° Fever	34 (14)	21 (13)	13 (16)	0.56
° Anorexia	73 (30)	46 (28)	27 (33)	0.50
° Weight loss	85 (35)	61 (38)	24 (29)	0.17
• Amount of weight loss, kg, median (IQR)	6 (4–8)^9^	6 (4–8)^7^	6 (5–7)^2^	0.80
° Morning stiffness	136 (83)^81^	102 (89)^47^	34 (69)^34^	**0.003**
• Duration, minutes, median (IQR)	60 (30–120)^59^	60 (30–120)^44^	60 (30–60)^15^	0.24
° Shoulder pain	212 (87)	150 (93)	62 (75)	**0.0001**
° Neck pain	63 (26)	45 (28)	18 (22)	0.30
° Lower back pain	55 (22)	35 (22)	20 (24)	0.66
° Pelvic girdle pain	188 (77)	133 (82)	55 (66)	**0.006**
° Pain in peripheral joints	108 (44)	73 (45)	35 (42)	0.67
° Swelling of peripheral joints	39 (16)	30 (19)	9 (11)	0.11
**Laboratory values**				
° ESR, mm/h, median (IQR)	46 (29.75–66.25)^37^	47 (35.5–66.25)^14^	30.5 (14.75–66.25)^23^	**0.007**
° CRP, mg/L, median (IQR	31.0 (9.325–67.975)^1^	36.0 (14.4–73.7)^1^	15.8 (2.55–60.95)	**0.0003**
° Hemoglobin, g/dL, median (IQR)	12.65 (11.4–13.68)^7^	12.4 (11.3–13.5)^5^	13.0 (11.9–14.1)^2^	**0.03**
Alkaline phosphatase, U/L, median (IQR)	100.5 (76.25–184.75)^31^	134 (81.25–210.25)^28^	85.5 (69.75–119.25)^3^	**<0.0001**

CRP, C-reactive protein; ESR, erythrocyte sedimentation rate; IQR, interquartile range; n, number; no., number; PET/CT, positron emission tomography/computed tomography; PMR, polymyalgia rheumatica. Number of missing values are reported in superscript. The bolded *p*-values are the *p*-values that are significant.

When considering all joints involved, FDG uptake was more frequently symmetrical in PMR patients compared to non-PMR patients (43.8 vs. 14.5%, *p* < 0.0001) (Supplementary Table 2). Also in each joint separately the FDG uptake was significantly more often symmetrical.

**FIGURE 2:**
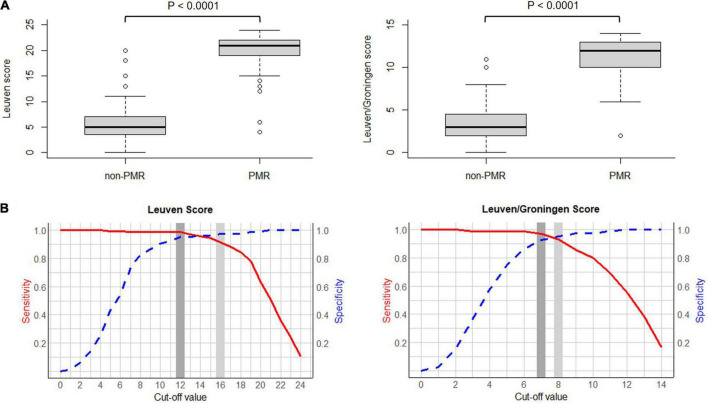
**(A)** Boxplot of the Leuven score (left) and the Leuven/Groningen score (right) in PMR patients vs. non-PMR patients. **(B)** Curve of the sensitivity and specificity of the Leuven score (left) and the Leuven/Groningen score (right) with variable cut-offs. Legend: light gray, predetermined cut-off value; dark gray, optimal cut-off value determined by the Youden index. Abbreviations: PMR, polymyalgia rheumatica.

The Leuven and Leuven/Groningen scores showed excellent interrater agreement (ICC 0.90 and 0.88, respectively). Both the Leuven and the Leuven/Groningen scores were significantly higher in PMR patients compared to non-PMR patients (21 [IQR 19–22] vs. 5 [IQR 3.5–7], *p* < 0.0001 and 12 [IQR 10–13] vs. 3 [IQR 2–4.5], *p* < 0.0001, respectively) (Figure 2). Both scores had an excellent and comparable diagnostic accuracy in the ROC analysis (AUC 0.986 [95% CI 0.971–1.000] and 0.980 [95% CI 0.963–0.997], respectively) (Table 2 and Figure 2 and Supplementary Figure 1). The *Leuven score* provided a sensitivity of 91.4% [95% CI 85.9–95.2%], a specificity of 97.6% [95% CI 91.6–99.7%] and an accuracy of 93.5% [95% CI 89.6–96.2%] at its predefined cut-off point of 16 with a positive LR of 37.9 [95% CI 9.6–149.2] and a negative LR of 0.09 [95% CI 0.05–0.15]. Based on the current cohort, the optimal cut-off point determined by the Youden index was 12. With this cut-off point, the sensitivity, specificity, accuracy, positive and negative LR were 98.8% [95% CI 95.6–99.9%], 95.2% [95% CI 88.1–98.7%], 97.6% [95% CI 95% CI 94.7–99.1%], 20.5 [95% CI 7.9–53.3], and 0.01 [95% CI 0.003–0.05], respectively. Four non-PMR patients had a Leuven score above the cut-off of 12: rotator cuff tendinopathy, rotator cuff tendinopathy combined with osteoarthritis of the lower back, systemic lupus erythematosus and Whipple’s disease in one patient each. The *Leuven/Groningen* score had a sensitivity of 93.2% [95% CI 88.2–96.6%], specificity of 95.2% [95% CI 88.1–98.7%], and accuracy of 93.9% [95% CI 90.1–96.5%] at its pre-specified cut-off point of 8 with a positive LR of 19.3 [95% CI 7.4–50.4] and a negative LR of 0.07 [95% CI 0.04–0.13]. The optimal cut-off point determined by the Youden index was 7 in the current cohort. With this cut-off point, the sensitivity, specificity, accuracy, positive and negative LR were 96.9% [95% CI 92.9–99.0%], 92.8% [95% CI 84.9–97.3%], 95.5% [95% CI 92.1–97.7%], 13.4 [95% CI 6.2–29.0], and 0.03 [95% CI 0.01–0.08]. Six non-PMR patients had a score of 7 or more with the following diagnoses: rotator cuff tendinopathy, systemic lupus erythematosus, Whipple’s disease, osteoarthritis, fibromyalgia and infection-related arthralgia in one patient each. A sensitivity analysis excluding patients with only non-AC images yielded similar results (Supplementary Table 3).

**TABLE 2:** Diagnostic accuracy of Leuven and Leuven/Groningen score.

Score	Sensitivity (95% CI)	Specificity (95% CI)	Accuracy (95% CI)	LR + (95% CI)	LR- (95% CI)	AUC (95% CI)
**Leuven score**						
Cut-off 16	91.4% (85.9–95.2%)	97.6% (91.6–99.7%)	93.5% (89.6–96.2%)	37.9 (9.6–149.2)	0.09 (0.05–0.15)	0.986 (0.971–1.000)
Cut-off 12	98.8% (95.6–99.9%)	95.2% (88.1–98.7%)	97.6% (94.7–99.1%)	20.5 (7.9–53.3)	0.01 (0.003–0.05)	
**Leuven/Groningen score**						
Cut-off 8	93.2% (88.2–96.6%)	95.2% (88.1–98.7%)	93.9% (90.1–96.5%)	19.3 (7.4–50.4)	0.07 (0.04–0.13)	0.980 (0.963–0.997)
Cut-off 7	96.9% (92.9–99.0%)	92.8% (84.9–97.3%)	95.5% (92.1–97.7%)	13.4 (6.2–29.0)	0.03 (0.01–0.08)	

95% CI, 95% confidence interval. AUC, area under the curve; LR, likelihood ratio.

## Discussion

This is the first study to compare and validate the diagnostic accuracy of the Leuven score and the Leuven/Groningen score for the diagnosis of PMR in a large real-life cohort. Our group developed the Leuven score in 2018 and found a sensitivity of 85.1% and specificity of 87.5% at a cut-off point of 16 (4). The retrospective study of van der Geest et al., in which several PET algorithms and scores were compared, yielded a comparable diagnostic accuracy of the Leuven score with a sensitivity of 89.7% and a specificity of 84.2% at the same cut-off point (14). In this study, we observed a higher diagnostic accuracy with a sensitivity of 91.4% and a specificity of 97.6%. Van der Geest et al. adapted the Leuven score to a more concise score, which provided a sensitivity of 89.7% and a specificity of 84.2% at the optimal cut-off point of 8 (14). This study confirms these findings with a sensitivity of 93.2% and a specificity of 95.2% for the Leuven/Groningen score. Thus, we found an excellent and comparable diagnostic accuracy for both scores with AUC 0.986 and 0.980, respectively, which are the best diagnostic accuracy results for a PET algorithm or score for PMR reported to date. However, we mainly want to emphasize the comparable performance of the Leuven and the Leuven/Groningen score. Since the Leuven/Groningen score only requires evaluation of 7 anatomic sites instead of 12, it may be easier to implement and apply in routine clinical practice.

Since this cohort is much larger than the studies in which the Leuven score and the Leuven/Groningen score were developed (99 and 58 patients, respectively) (4, 14), we determined the optimal cut-off points in the current cohort. We found an optimal cut-off point of 12 and 7, respectively, which were both lower than the initial cut-off points. This resulted in a sensitivity and specificity of 98.8% and 95.2% for the Leuven score and 96.9% and 92.8% for the Leuven/Groningen score, respectively. The predefined and newly determined cut-off points should ideally be compared in a large prospective cohort to determine the definitive cut-off. Several cut-off points may be applied depending on the preference for a higher sensitivity or specificity. All cut-off points between 10 and 16 for the Leuven score and a cut-off point of 7 or 8 for the Leuven/Groningen score had a sensitivity and specificity for PMR over 90%.

In this study, PMR patients were significantly older and had a more pronounced inflammatory response. The symptom duration was longer in non-PMR patients probably due to a considerable amount of patients with a mechanical cause in this group. PMR patients more frequently reported shoulder and pelvic girdle pain and morning stiffness. In addition, we observed more frequently a symmetrical articular and periarticular FDG uptake in PMR patients compared to non-PMR patients. Since the diagnostic accuracy of the Leuven and Leuven/Groningen score is already excellent, consideration of the symmetry would make the scores more complex without a large increase in diagnostic yield.

This study confirms that FDG-PET may be an excellent diagnostic tool for PMR. Additionally, FDG-PET may contribute to the diagnosis of common mimics of PMR. In our cohort, PET revealed an alternative diagnosis in 8 of the 83 (9.6%) non-PMR patients.

Strengths of our study include the large cohort, a control group consisting of patients with PMR-like disorders who had a similar clinical presentation, the fact that PET imaging was performed prior to the initiation of glucocorticoids and the assessment of interrater agreement. This study also has several limitations. First, part of the FDG-PET scans were performed on a stand-alone PET system with lower resolution and sensitivity compared with current PET-CT systems. In addition, PET images without CT-scan preclude attenuation correction, so the mean liver uptake is an assessment by the reader. However, sensitivity analysis excluding these PET scans found similar results. Second, the interrater agreement was only assessed in the first 20 patients. Third, we did not assess PET images quantitatively as we aimed to validate the Leuven and the Leuven/Groningen score. These scores used a semi-quantitative method with visual scores intended for application in clinical practice. Compared to quantitative methods, they require less experience and are less time-consuming. In addition, selection bias due to the retrospective design could have potentially influenced the results. Fifth, the clinicians responsible for the diagnosis of PMR had access to the PET images, since it was performed as part of routine clinical practice. This could result in circular reasoning and increased diagnostic accuracy. However, PMR-PET scores were not specified in the PET report and the final diagnosis was based on all available diagnostic information. In addition, the two nuclear specialists who re-evaluated the PET images, were blinded for all patient information. Finally, since all patients were diagnosed and followed in a general internal medicine department, the non-PMR group contained only a limited number of patients with rheumatic diseases, which could explain the high specificity of the Leuven and Leuven/Groningen score.

In conclusion, the Leuven score and the Leuven/Groningen score provided an excellent and comparable diagnostic accuracy in a real-life cohort. Due to the lower number of anatomic sites that needs to be evaluated, the Leuven/Groningen score may be easier to apply in clinical practice. The optimal cut-off points were 12 for the Leuven score and 7 for the Leuven/Groningen score, but require further validation in a large prospective cohort. Further imaging studies should address if these scores can be used for disease monitoring.

## Data availability statement

The raw data supporting the conclusions of this article will be made available by the authors, without undue reservation.

## Ethics statement

The studies involving human participants were reviewed and approved by the Ethical Committee of the University Hospitals Leuven. Written informed consent for participation was not required for this study in accordance with the national legislation and the institutional requirements.

## Author contributions

LM: conceptualization, methodology, validation, investigation, formal analysis, writing – original draft, writing – review and editing, and visualization. LB: conceptualization, methodology, validation, investigation, and writing – review and editing. AB and KV: conceptualization, methodology, investigation, and writing – review and editing. MV: investigation and writing – review and editing. SV and DB: conceptualization, writing – review and editing, and supervision. All authors contributed to the article and approved the submitted version.
