# Supplementary material for: Diagnostic accuracy and validation of 18F-fluorodeoxyglucose positron emission tomography scores in a large cohort of patients with polymyalgia rheumatica
Source: Front Med (Lausanne). 2022 Sep 21;9:1026944. doi: 10.3389/fmed.2022.1026944 (PMC9533121; doi:10.3389/fmed.2022.1026944)
Supplement: Supplementary file 1 [file Data_Sheet_1.docx]

Supplementary Material

# Supplementary Figures and Tables

## Supplementary Figures

**
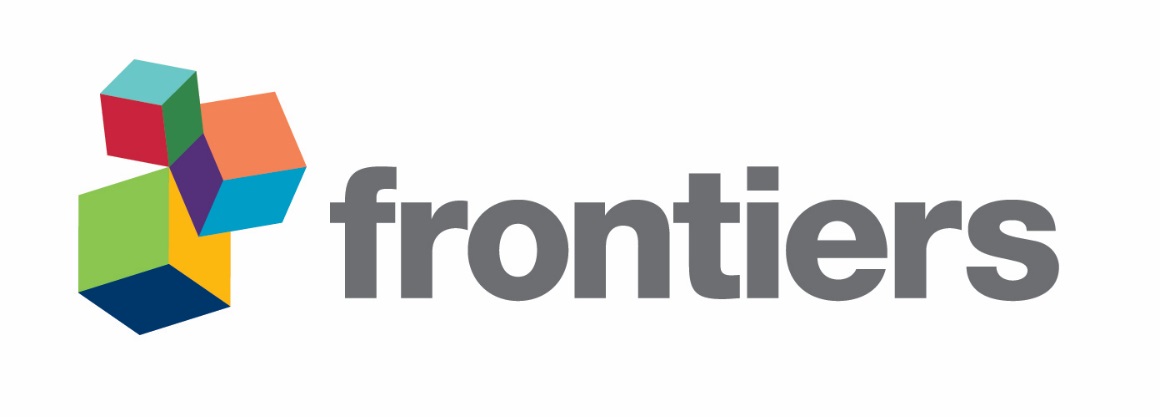
**

**Supplementary Figure 1:** Receiver operating characteristic curve of the Leuven score (in red) and the Leuven/Groningen score (in blue)

*Abbreviations: AUC, area under the curve*

**
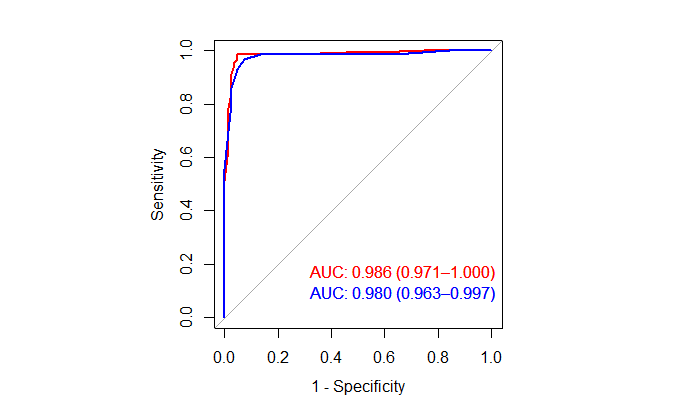
**

## Supplementary tables

**Supplementary Table 1:** Alternative diagnoses in non-PMR patients

| **Alternative diagnosis** | **N (%)** | **Number of PET-scans contributive to the alternative diagnosis (%)** |
| --- | --- | --- |
| Osteoarthritis | 14 (17) | 0 (0) |
| Rotator cuff tendinopathy | 2 (2) | 0 (0) |
| Tendomyalgia | 5 (6) | 0 (0) |
| Fibromyalgia | 12 (14) | 0 (0) |
| Rotator cuff tendinopathy + tendomyalgia | 2 (2) | 0 (0) |
| Rotator cuff tendinopathy + osteoarthritis | 3 (3) | 0 (0) |
| Rotator cuff tendinopathy + viral syndrome with spontaneous resolution | 1 (1) | 0 (0) |
| Tendomyalgia + carpal tunnel syndrome | 1 (1) | 0 (0) |
| Osteoarthritis + neuropathic pain | 1 (1) | 0 (0) |
| Loosening of shoulder prosthesis | 1 (1) | 0 (0) |
| Spontaneous resolution | 12 (15) | 0 (0) |
| Rheumatoid arthritis | 2 (2) | 0 (0) |
| Polyarteritis nodosa | 1 (1) | 1 (1) |
| IgG4-related disease | 1 (1) | 0 (0) |
| Systemic lupus erythematosus | 3 (4) | 0 (0) |
| Dermatomyositis | 1 (1) | 1 (1) |
| Sjögren’s syndrome | 1 (1) | 0 (0) |
| Microscopic polyangiitis | 1 (1) | 0 (0) |
| Amyotrophic lateral sclerosis | 1 (1) | 0 (0) |
| Liver abscess | 1 (1) | 1 (1) |
| Cholangitis | 1 (1) | 1 (1) |
| Septic arthritis | 1 (1) | 0 (0) |
| Infection-related arthralgia | 1 (1) | 0 (0) |
| Whipple’s disease | 1 (1) | 0 (0) |
| Paraneoplastic syndrome | 5 (6) | 4 (5) |
| Multiple myeloma | 1 (1) | 0 (0) |
| Side effect of medication | 1 (1) | 0 (0) |
| No diagnosis | 6 (7) | 0 (0) |

*Abbreviations: Ig, immunoglobulin; N, number; PMR, polymyalgia rheumatica*

**Supplementary Table 2:** Symmetry in the FDG uptake

| Joint | PMR (n = 162) | Non-PMR (n = 83) | P-value |
| --- | --- | --- | --- |
| Sternoclavicular joint  Number of patients with FDG uptake  0 and 1  1 and 2  0 and 2  Symmetrical uptake (both 1 or 2) | 150 (92.6%)  12 (7.4%)  32 (21.3%)  1 (0.7%)  105 (70.0%) | 35 (42.2%)  20 (57.1%)  4 (11.4%)  1 (2.9%)  10 (28.6%) | **< 0.0001**  0.18  0.26  **< 0.0001** |
| Shoulder  Number of patients with FDG uptake  0 and 1  1 and 2  0 and 2  Symmetrical uptake (both 1 or 2) | 162 (100%)  1 (0.6%)  19 (11.7%)  0 (0%)  142 (87.7%) | 56 (67.5%)  20 (35.7%)  13 (23.2%)  2 (3.6%)  21 (37.5%) | **< 0.0001**  **0.04**  **0.02**  **< 0.0001** |
| Ischial tuberosity  Number of patients with FDG uptake  0 and 1  1 and 2  0 and 2  Symmetrical uptake (both 1 or 2) | 158 (97.5%)  9 (5.7%)  17 (10.8%)  2 (1.3%)  130 (82.3%) | 30 (36.1%)  15 (50.0%)  2 (6.7%)  0 (0%)  13 (43.3%) | **< 0.0001**  0.50  0.54  **< 0.0001** |
| Greater trochanter  Number of patients with FDG uptake  0 and 1  1 and 2  0 and 2  Symmetrical uptake (both 1 or 2) | 160 (98.7%)  2 (1.3%)  27 (16.9%)  0 (0%)  131 (81.9%) | 73 (88.0%)  14 (19.2%)  12 (16.4%)  1 (1.4%)  46 (63.0%) | **< 0.0001**  0.93  0.14  **0.002** |
| Hip  Number of patients with FDG uptake  0 and 1  1 and 2  0 and 2  Symmetrical uptake (both 1 or 2) | 161 (99.4%)  2 (1.2%)  13 (8.1%)  1 (0.6%)  145 (90.1%) | 35 (42.2%)  18 (51.4%)  6 (17.1%)  2 (5.7%)  9 (25.7%) | **< 0.0001**  0.10  **0.02**  **< 0.0001** |
| Symmetrical uptake in all joints (both 0,1 or 2) | 71 (43.8%) | 12 (14.5%) | **< 0.0001** |

*Abbreviations: FDG, fluorodeoxyglucose; n, number; PMR, polymyalgia rheumatica*

**Supplementary Table 3:** Diagnostic accuracy of the Leuven and Leuven/Groningen score excluding patients with only non-AC images (PET scan)

| Score | Sensitivity | Specificity | LR+ | LR- | AUC |
| --- | --- | --- | --- | --- | --- |
| Leuven Score  Cut-off 16  Cut-off 12 | 85.1% (74.3-92.6%)  98.5% (92.0-100.0%) | 100% (93.2-100.0%)  100% (93.2-100.0%) | infinity  infinity | 0.15 (0.08-0.26)  0.02 (0.00-0.10) | 0.992 (0.976-1.000) |
| Leuven/Groningen Score  Cut-off 8  Cut-off 7 | 89.6% (79.7-95.7%)  94.0% (85.4-98.3%) | 96.2% (86.8-99.5%)  94.2% (84.1-98.8%) | 23.3 (6.0-90.8)  16.3 (5.4-49.0) | 0.11 (0.05-0.22)  0.06 (0.02-0.16) | 0.984 (0.963-1.000) |
